# Melatonin suppresses cancer cell proliferation, DNA repair and expression of the oncogene TRIP13

**DOI:** 10.1038/s41420-025-02788-z

**Published:** 2025-10-27

**Authors:** Wenqing Liu, Ans M. M. van Pelt, Geert Hamer

**Affiliations:** 1 Reproductive Biology Laboratory, Center for Reproductive Medicine, Amsterdam UMC, University of Amsterdam, Amsterdam, The Netherlands; 2 Amsterdam Reproduction and Development Research Institute, Amsterdam, the Netherlands

**Keywords:** Cell death, Oncogenes

## Abstract

Non-small cell lung cancer (NSCLC) continues to be a global health challenge, with limited treatment options and a high mortality rate. We recently found that lung cancer cells that express more genes that are typically restricted to the germline (germ cell cancer genes, GC genes) repair DNA double-strand breaks more rapidly, show higher rates of proliferation, and are more resistant to ionizing radiation, compared to cells that express fewer GC genes. Moreover, we found that the gene encoding TRIP13 (thyroid hormone receptor interactor 13) plays a significant role in this malignant phenotype. Here, we demonstrate that melatonin (MT), a hormone synthesized in the pineal gland, downregulates the expression of TRIP13, particularly in lung cancer cells with high expression of TRIP13. Moreover, this downregulation of TRIP13 by MT further inhibits the DNA repair proteins RAD51 and XRCC5, thereby impairing DNA repair via homologous recombination and non-homologous end joining. We further found that the melatonin receptor 1B (MTNR1B), rather than melatonin receptor 1 A (MTNR1A), is essential for MT mediated TRIP13 downregulation. Because we also found that treatment with MT still decreases cell proliferation of TRIP13-KO cells, combining MT with the TRIP13 inhibitor DCZ0415 would likely have an additive anti-proliferative therapeutic effect in the treatment of NSCLC.

## Introduction

Treatment of lung adenocarcinoma (LUAD) and lung squamous cell carcinoma (LUSC), the two major subtypes of non-small cell lung cancer (NSCLC), remains a significant challenge in oncology [1]. As a leading cause of cancer-related mortality worldwide, NSCLC demands a nuanced understanding and targeted therapeutic approaches. The hormone melatonin (N-acetyl-5-methoxytryptamine, MT), traditionally recognized for its involvement in circadian rhythm regulation, has gained attention for its anticancer properties in various malignancies [2]. In recent years, MT has become a subject of intense interest in cancer research due to its diverse physiological roles and its potential as a modulator of tumorigenic processes [3]. In mammals, MT typically regulates survival signaling and tumor progression through two G protein-coupled membrane receptors, namely MT receptor 1 A (MTNR1A) and MT receptor 1B (MTNR1B) [4]. In NSCLC research, MT has emerged as a promising modulator of tumorigenic processes, including cell proliferation, apoptosis, and cytotoxicity response [5–7].

Many genes have been identified as drivers of the transition process from healthy cells to cancer cells. Such oncogenes contribute to the acquisition of cancer-specific hallmarks, such as uncontrolled cell proliferation, cell migration, genome instability, and aberrant apoptosis regulation [8]. Targeting these hallmark processes is effectively used by many current cancer therapies. However, because these processes are also used by many healthy cells, most cancer treatments cause severe side effects. We recently found that tumors express many genes that are normally restricted to the germline (germ cell cancer genes, GC genes) [9, 10]. Moreover, expression of such germline genes appears to increase the malignancy of cancer cells [11, 12]. We recently found that lung cancer cells expressing more GC genes can repair DNA double-strand breaks (DSBs) more rapidly, show higher rates of proliferation, and are more resistant to ionizing radiation. Importantly, the thyroid hormone receptor interactor 13 (TRIP13) appeared to strongly enhance this phenotype [13].

TRIP13 is an AAA-ATPase involved in various cellular processes, with high expression during embryogenesis and in testicular tissue [14–16]. It plays a crucial role in mitosis, particularly in the spindle assembly checkpoint and metaphase-to-anaphase transition, as well as in meiosis, regulating checkpoints and homologous recombination (HR) during the first meiotic G2/prophase stage [17]. TRIP13 is necessary for proper chromosomal pairing and resolution of recombination intermediates in somatic cells, influencing the choice between HR and non-homologous end-joining (NHEJ) pathways [18]. It is involved in the resolution of recombination intermediates that arise during HR, promoting the proper disassembly of DNA joint molecules and ensuring the accurate exchange of genetic information between homologous chromosomes during meiosis [19]. TRIP13 also influences the choice between different pathways of HR, and many studies suggest that TRIP13 could contribute to the establishment of inter-homolog biased HR [20–23]. Understanding the diverse functions of TRIP13 is crucial for unraveling its implications in physiological and pathological conditions, particularly in cancer, where disruptions in cell division and DNA repair can lead to tumorigenesis [24]. We previously found that TRIP13 is endogenously expressed in NSCLC, and that inhibiting TRIP13 increases the effectiveness of treatment with ionizing radiation [13, 25]. Here we find that MT, through MTNR1B rather than MTNR1A, downregulates TRIP13 expression and, further downstream, the DSB repair protein RAD51. This gives a novel mechanism in which MT, through downregulation of TRIP13, slows down aberrant DNA repair and proliferation of cancer cells.

## Results

### Melatonin inhibits NSCLC cell proliferation

**Fig. 1:**
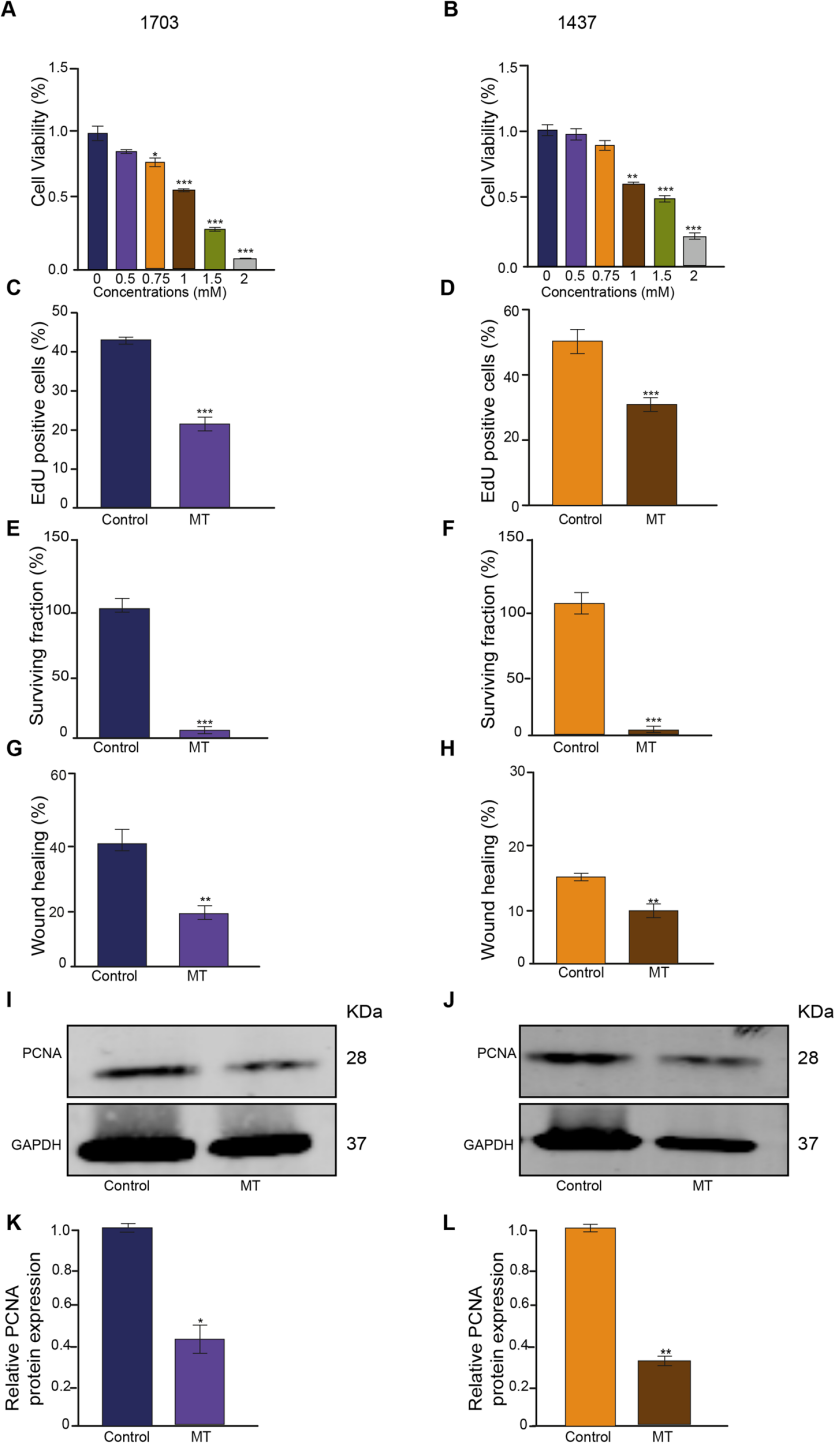
Melatonin (MT) inhibits NSCLC cell proliferation and wound healing. Dose-dependent decrease of cell viability upon treatment with MT in H1703 cells (**A**) and H1437 cells (**B**) as detected by Alamar Blue (*n* = 3). **C** Quantification of proliferation (with or without 1 mM MT) of H1703 cells measured by EdU incorporation (*n* = 9). **D** Quantification of proliferation (with or without 1 mM MT) of H1437 cells measured by EdU incorporation (*n* = 9). **E** Clonogenic survival rate (with or without 1 mM MT) of H1703 cells (*n* = 3). **F** clonogenic survival rate (with or without 1 mM MT) of H1437 cells (*n* = 3). **G** Wound healing rate (with or without 1 mM MT) of H1703 cells. (*n* = 3). **H** Wound healing rate (with or without 1 mM MT) of H1437 cells (*n* = 3). **I**, **J** Expression of PCNA levels (with or without 1 mM MT) in the H1703 cells and H1437 cells. **K**, **L** PCNA protein levels quantified relative to GAPDH in H1703 and H1437 cells (with or without 1 mM MT), (*n* = 3). *p < 0.05. **p < 0.01. ***p < 0.001.

To test the biological effects of MT on NSCLC cells, we chose two NSCLC cell lines, H1703 and H1437. We previously described that both these cell lines express high levels of TRIP13; however, H1703 expresses more GC genes than H1437 [13]. These cells were treated with different concentrations of MT for 48 hours. Alarm Blue was used to detect cell viability. As shown in Fig. 1A, B, the viability of both NSCLC cell types was inhibited in a dose-dependent manner after the addition of MT (Fig. 1A, B). To further explore the anticancer effects of MT, we chose to use 1 mM MT for 48 h.

To investigate the effect of MT on cell proliferation, we stained cells, with and without MT treatment, with DNA synthesis marker 5-ethynyl-2’-deoxyuridine (EdU) (Fig. 1C, D; Supplementary Fig. S1A, B). MT treatment inhibited cell proliferation compared with that in the control group (Fig. 1C, D; Supplementary Fig. S1A, B).

In addition, also colony formation assays in response to MT were performed to quantify cell proliferation. NSCLC cells treated with MT showed a significant decrease in surviving fraction after treatment with MT for 48 h, indicating that this compound inhibits cell proliferation (Fig. 1E, F; Supplementary Fig. S1C, D).

In order to assess the impact of MT on the NSCLC cell growth and migration, cells were treated with or without 1 mM MT for 24 h and evaluated using the wound healing assay to investigate the effects of MT on their migration ability (Fig. 1G, H; Supplementary Fig. S1E, F). Wound healing rates were reduced in the presence of MT for both H1703 and H1437 cells (Fig. 1G, H; Supplementary Fig. S1E, F).

Finally, after 48 h exposure to 1 mM MT, we quantified the proliferation marker PCNA using Western blot analysis. PCNA appeared to be decreased in response to MT in both the H1703 and H1437 cells (Fig. 1I–L). Together, these results show that MT prevents cell proliferation in high TRIP13-expressing NSCLC cell lines.

### The expression of TRIP13 correlates with LUAD patient survival

**Fig. 2:**
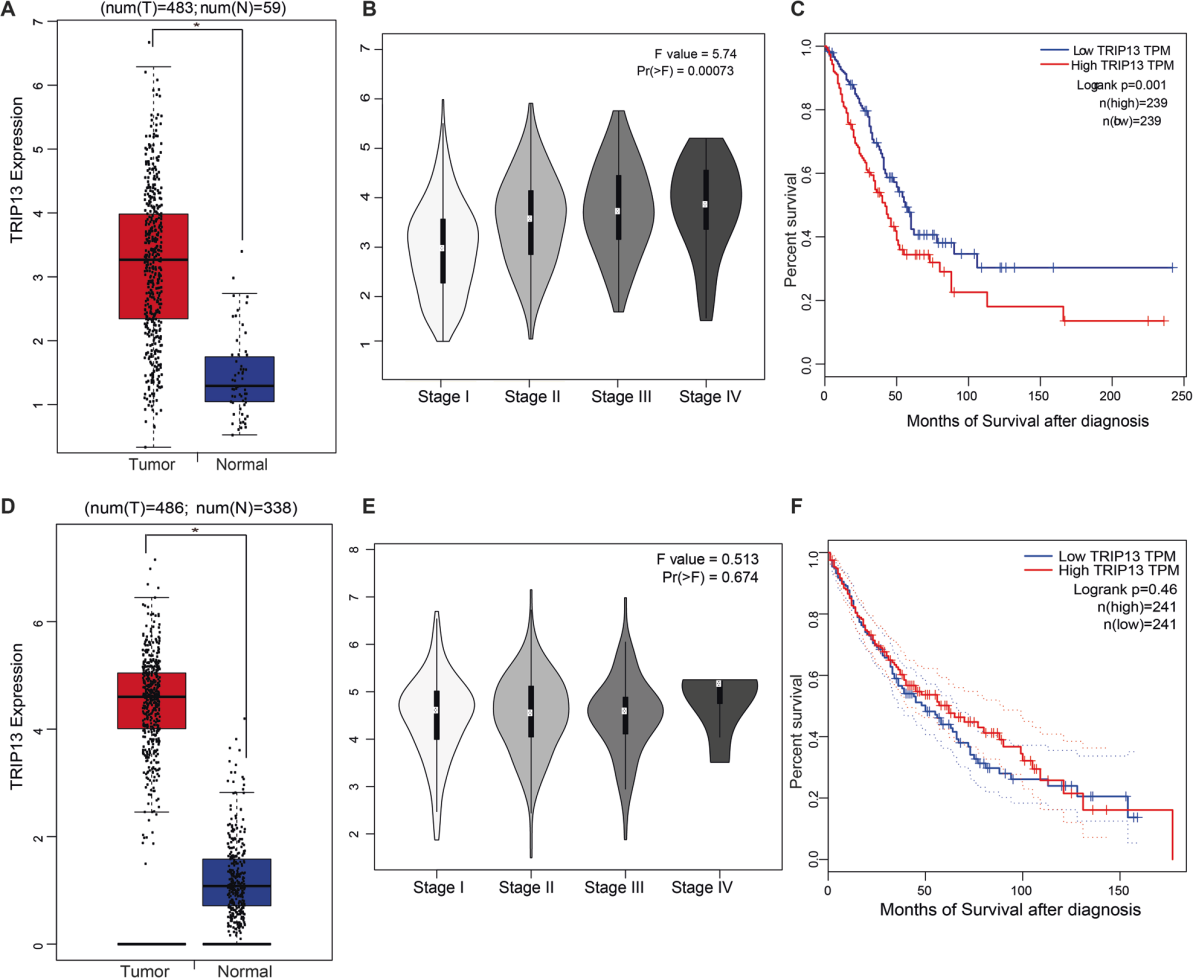
Expression of TRIP13 in LUAD and LUSC and its correlation with LUAD patient survival. **A** TRIP13 expression was upregulated in LUAD (*n* = 483) compared with normal lung tissues (*n* = 59). **B** Expression levels of TRIP13 in LUAD based on individual cancer stages. **C** Kaplan–Meier Plot of the survival rate of LUAD patients with high or low levels of TRIP13 (median, *p* = 0.001). **D** TRIP13 expression was upregulated in LUSC (*n* = 486) compared with normal lung tissues (*n* = 338). **E** Expression levels of TRIP13 in LUSC based on individual cancer stages. **F** Kaplan–Meier plot of the survival rate of LUSC patients with high or low levels of TRIP13. All data shown are based on transcriptomic (mRNA) analysis from the public TCGA dataset.

Analyses using The Cancer Genome Atlas (TCGA) databases (http://gepia.cancer-pku.cn/) showed that TRIP13 mRNA expression was higher in LUAD than in healthy lung tissues (Fig. 2A). For LUAD, TRIP13 mRNA expression significantly increased with increasing LUAD grade (Fig. 2B), and Kaplan–Meier survival analysis showed that LUAD patients with high TRIP13 had lower overall survival (OS) (Fig. 2C). Also for LUSC, the TCGA databases showed that TRIP13 expression appeared significantly higher in LUSC than in healthy lung tissues in RNA level (Fig. 2D). TRIP13 mRNA expression significantly increased from LUSC Stage III to Stage IV (Fig. 2E), However, Kaplan–Meier survival analysis showed that LUSC patients with high TRIP13 did not display an increased OS (Fig. 2F). Together, these results suggest that high expression of TRIP13 mRNA promotes the malignant progression of LUAD and LUSC and leads to a poor prognosis for patients with LUAD.

### Potential involvement of MTNR1B (MT2) in TRIP13 downregulation by MT in NSCLC cells

**Fig. 3:**
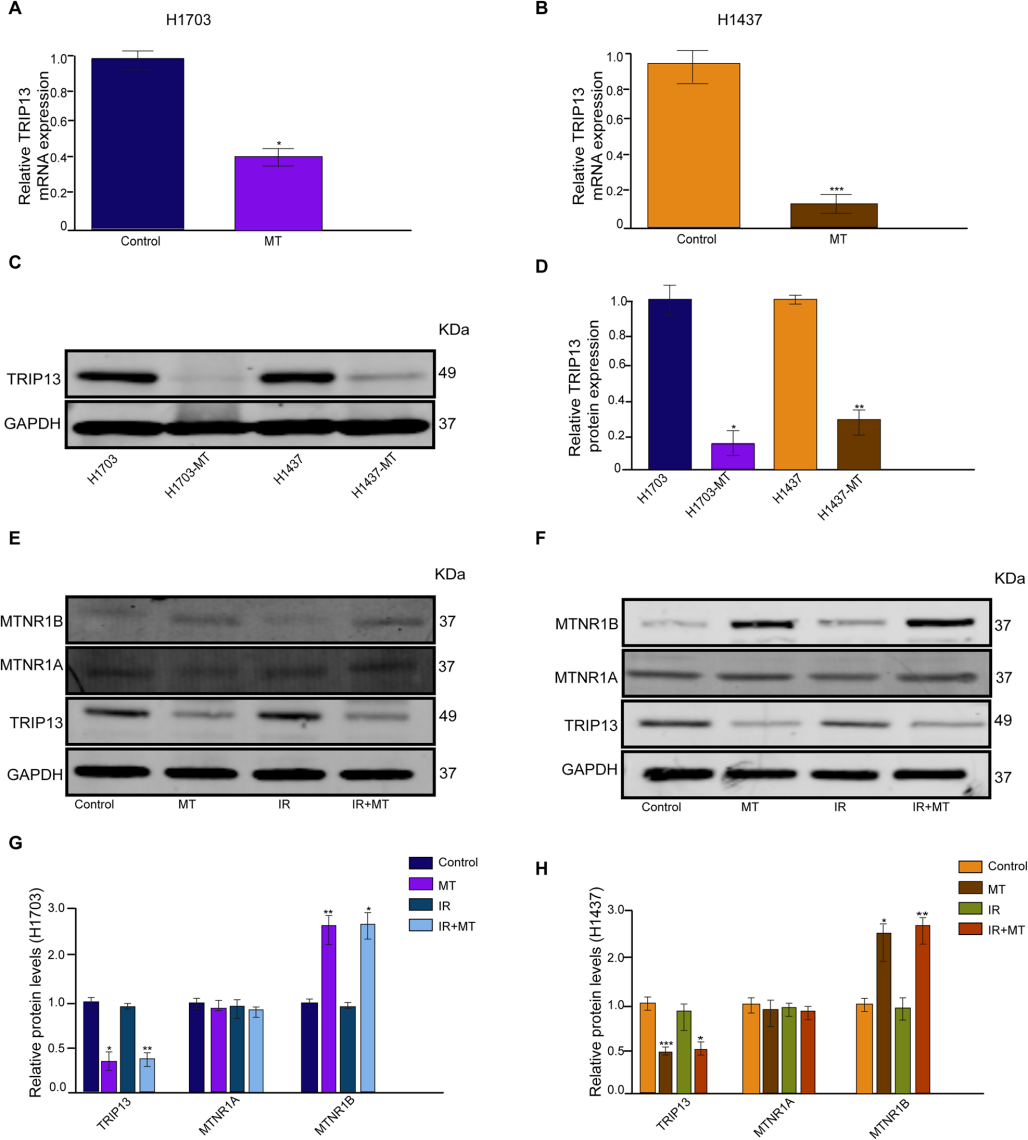
Melatonin (MT) treatment is associated with increased MTNR1B expression and reduced TRIP13 levels. **A** Expression of TRIP13 mRNA in H1703 cells with or without MT treatment (*n* = 3). **B** Expression of TRIP13 mRNA in H1437 cells with or without MT treatment (*n* = 3). **C** Expression of TRIP13 levels in H1703 and H1437 cells with or without MT treatment. **D** TRIP13 protein levels quantified relative to GAPDH in H1703 and in H1437 cells with or without MT treatment (*n* = 3). **E** Expression of TRIP13, MTNR1A, and MTNR1B proteins of control, MT, IR, and IR combined with MT treated in the H1703 cells. **F** Expression of TRIP13, MTNR1A, and MTNR1B proteins of control, MT, IR, and IR combined with MT treated H1437 cells. **G** Quantification of TRIP13, MTNR1A, and MTNR1B protein levels relative to GAPDH of control, MT, IR, and IR + MT treated H1703 cells (*n* = 3). **H** Quantification of TRIP13, MTNR1A, and MTNR1B protein levels relative to GAPDH of control, MT, IR, and IR + MT treated H1437 cells (*n* = 3). *p < 0.05. **p < 0.01. ***p < 0.001.

To investigate the relationship between MT and *TRIP13* in NSCLC cells, first, we treated cells with MT and examined the RNA and protein expression of *TRIP13*. Compared to the control group (0 mM MT), treatment with MT resulted in a significant decrease in the expression of *TRIP13* at both the RNA (Fig. 3A, B) and protein levels (Fig. 3C, D) for both cell lines. In general, the effects of MT are mediated through two G-protein-coupled membrane receptors, namely MT receptor 1 (MTNR1A) and MT receptor 2 (MTNR1B) [26]. To determine which melatonin receptor may be mediating TRIP13 expression, Western blot analysis was conducted to detect the expression of MTNR1A and MTNR1B. In comparison to the control group, cells treated with MT showed a reduction in TRIP13 protein expression and a significant upregulation of MTNR1B expression (Fig. 3E–H). However, no significant change in MTNR1A expression was observed (Fig. 3E–H).

To investigate whether ionizing radiation (IR) induces changes in MT receptor expression, we performed Western blot analysis to detect the MT receptors with and without a dose of 2 Gy (IR). After IR, there was no change in basal TRIP13 expression or in the MT receptors (Fig. 3E–H). IR given in combination with MT gave similar results to MT alone. Taken together, these observations suggest that the downregulation of TRIP13 by MT may be associated with MTNR1B upregulation rather than MTNR1A, with no change following 2 Gy of IR.

### Blockage of MTNR1B impairs melatonin’s ability to reduce cell proliferation and decrease TRIP13

**Fig. 4:**
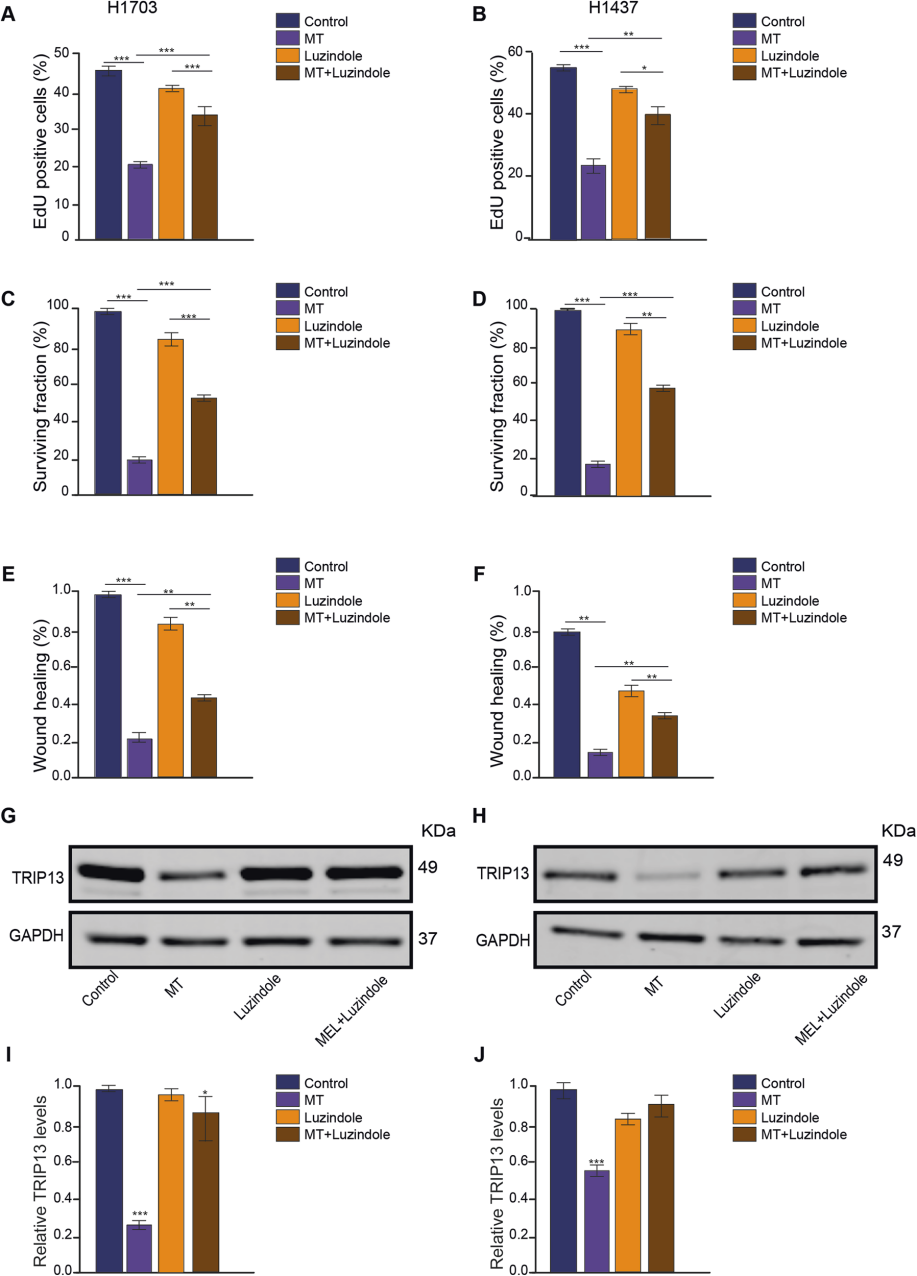
Blockage of MTNR1B impairs melatonin’s ability to reduce cell proliferation and TRIP13 expression. Quantification of the effect of control, MT, Luzindole, and MT+Luzindole treatments on **A** proliferation of H1703 cells measured by EdU incorporation (*n*=3). **B** proliferation of H1437 cells measured by EdU incorporation (*n* = 3). **C** clonogenic survival rate of H1703 cells (*n* = 3). **D** clonogenic survival rate of H1437 cells (*n* = 3). **E** Wound healing rate of H1703 cells. (*n* = 3). **F** Wound healing rate of H1437 cells. (*n* = 3). **G**, **H** Expression of TRIP13 protein in control, MT, Luzindole, and MT+Luzindole treated in the H1703 and H1437 cells. **I**, **J** TRIP13 protein levels quantified relative to GAPDH in control, MT, Luzindol,e and MT+Luzindole treated H1703 cells and H1437 cells (*n* = 3). *p < 0.05. **p < 0.01. ***p < 0.001.

To further confirm that MT exerts its effects through MTNR1B, cells were treated with MTNR1B inhibitor Luzindole [27], and we again performed the EdU assay to detect cell proliferation, but also added Luzindole. This assay showed that cells treated with MT showed significantly decreased cell proliferation. However, in combination with Luzindole the inhibitory effect of MT on cell proliferation was impaired (Fig. 4A, B; Supplementary Fig. S2A, B).

This was confirmed by clonogenic assays that showed that cells treated with MT displayed significantly decreased surviving fraction (Fig. 4C, D; Supplementary Fig. S2C, D). This decrease could be reduced by adding the MTNR1B inhibitor Luzindole during the treatment with MT, showing that the inhibitory effect of MT on long-term colony formation was impaired (Fig. 4C, D; Supplementary Fig. S2C, D).

To further investigate the effect of MT and MTNR1B inhibition on cell growth and migration, the wound healing assay was performed. Again, addition of Luzindole reduced the effect of MT (Fig. 4E, F; Supplementary Fig. S2E, F).

We next explored whether inhibition of the MT receptor MTNR1B would block the MT induced decrease in TRIP13. Western blot analysis showed that MT again reduced TRIP13 in both cell lines. This reduction was blocked by adding Luzindole, showing that MT decreases TRIP13 via the MTNR1B receptor (Fig. 4G–J).

**Fig. 5:**
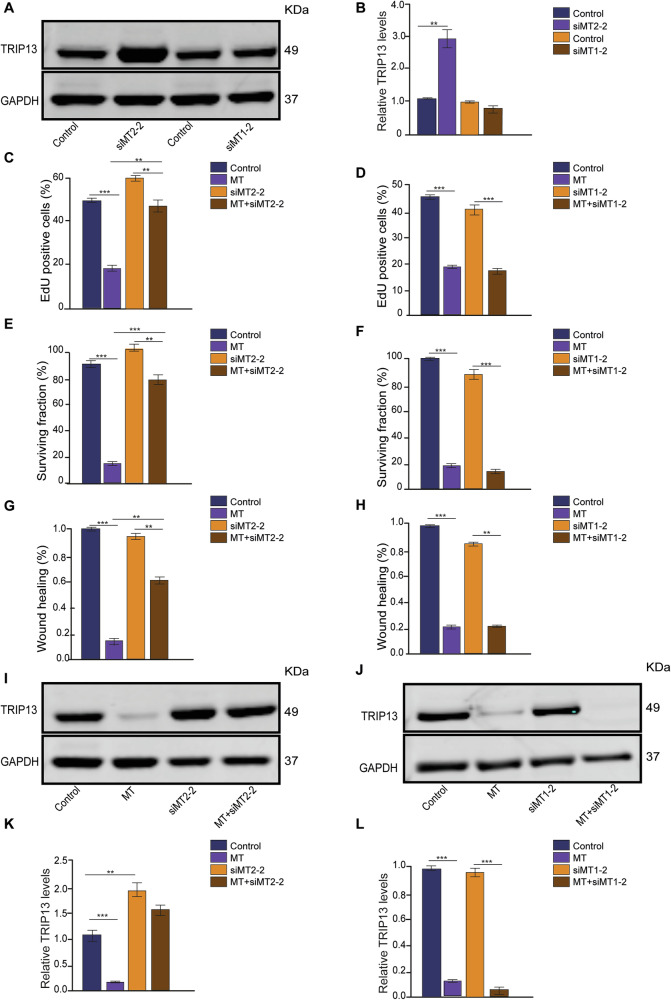
knock down of MTNR1B impairs melatonin’s ability to reduce cell proliferation and TRIP13 expression. **A** Expression of TRIP13 protein in control and siMT2-2, and control and siMT1-2 treated H1703 cells. **B** TRIP13 protein levels quantified relative to GAPDH in control and siMT2-2, and control and siMT1-2 treated H1703 cells (*n* = 3). **C** Quantification of the effect of control, MT, siMT2-2, and MT+siMT2-2 treatments on proliferation of H1703 cells measured by EdU incorporation (*n* = 3). **D** Quantification of the effect of control, MT, siMT1-2, and MT+siMT1-2 treatments on the proliferation of H1703 cells measured by EdU incorporation (*n* = 3). **E** Clonogenic survival rate of control, MT, siMT2-2, and MT+siMT2-2 treated H1703 cells (*n* = 3). **F** Clonogenic survival rate of control, MT, siMT1-2, and MT+siMT1-2 treated H1703 cells (*n* = 3). **G** Wound healing rate of control, MT, siMT2-2, and MT+siMT2-2 treated H1703 cells (*n* = 3). **H** Wound healing rate of control, MT, siMT1-2, and MT+siMT1-2 treated H1703 cells (*n* = 3). **I** Expression of TRIP13 protein in control, MT, siMT2-2, and MT+siMT2-2 treated H1703 cells. **J** Expression of TRIP13 protein in control, MT, siMT1-2, and MT+siMT1-2 treated H1703 cells. **K** TRIP13 protein levels quantified relative to GAPDH in control, MT, siMT2-2, and MT+siMT2-2 treated H1703 cells (*n* = 3). **L** TRIP13 protein levels quantified relative to GAPDH in control, MT, siMT1-2, and MT+siMT1-2 treated H1703 cells (*n* = 3). **p < 0.01. ***p < 0.001.

To further confirm that MT exerts its effects through MT2, H1703 cells were treated with MEL-1A-R siRNA or MEL-1B-R siRNA to generate MT1 and MT2 knockdown cell lines. Western blot analysis confirmed a reduction in MT1 and MT2 protein levels (Supplementary Fig. S3A and Supplementary Fig. S4A). Notably, TRIP13 expression increased following MT2 receptor knockdown, whereas MT1 receptor knockdown had no effect on TRIP13 expression (Fig. 5A, B). These findings further support our previous conclusion that TRIP13 downregulation occurs via MT2 rather than MT1.

We then performed an EdU assay to measure cell proliferation. This assay again showed that cells treated with MT displayed a significantly reduced cell proliferation. However, this inhibitory effect was only impaired in MT2 knockdown cells and not after MT1 knockdown (Fig. 5C, D; Supplementary Fig. S3B and Supplementary Fig. S4B). These findings were confirmed by clonogenic assays, which demonstrated a significant reduction in the surviving fraction of MT treated cells (Fig. 5E, F; Supplementary Fig. S3C and Supplementary Fig. S4C). This decrease was reduced by MT2 knockdown but not by MT1 knockdown, indicating that the inhibitory effect of MT on long-term colony formation was impaired by MT2 knockdown (Fig. 5E, F; Supplementary Fig. S3C and Supplementary Fig. S4C).

To further investigate the effects of MT and MT2 receptor knockdown on cell growth and migration, a wound healing assay was performed (Supplementary Fig. S3D and Supplementary Fig. S4D). Again, the results showed that MT2 receptor knockdown attenuated the inhibitory effect of MT (Fig. 5G, H).

We next explored whether a decrease in the MT receptor would block the MT-induced decrease in TRIP13 expression. Western blot analysis showed that MT reduced TRIP13 in both control cell lines (Fig. 5I–L). However, this reduction in TRIP13 was impaired by MT2 knockdown, while it remained unaffected in MT1 knockdown cells, demonstrating that MT decreases TRIP13 expression via the MTNR1B receptor (Fig. 5I–L).

### Melatonin inhibits HR and suppresses TRIP13

**Fig. 6:**
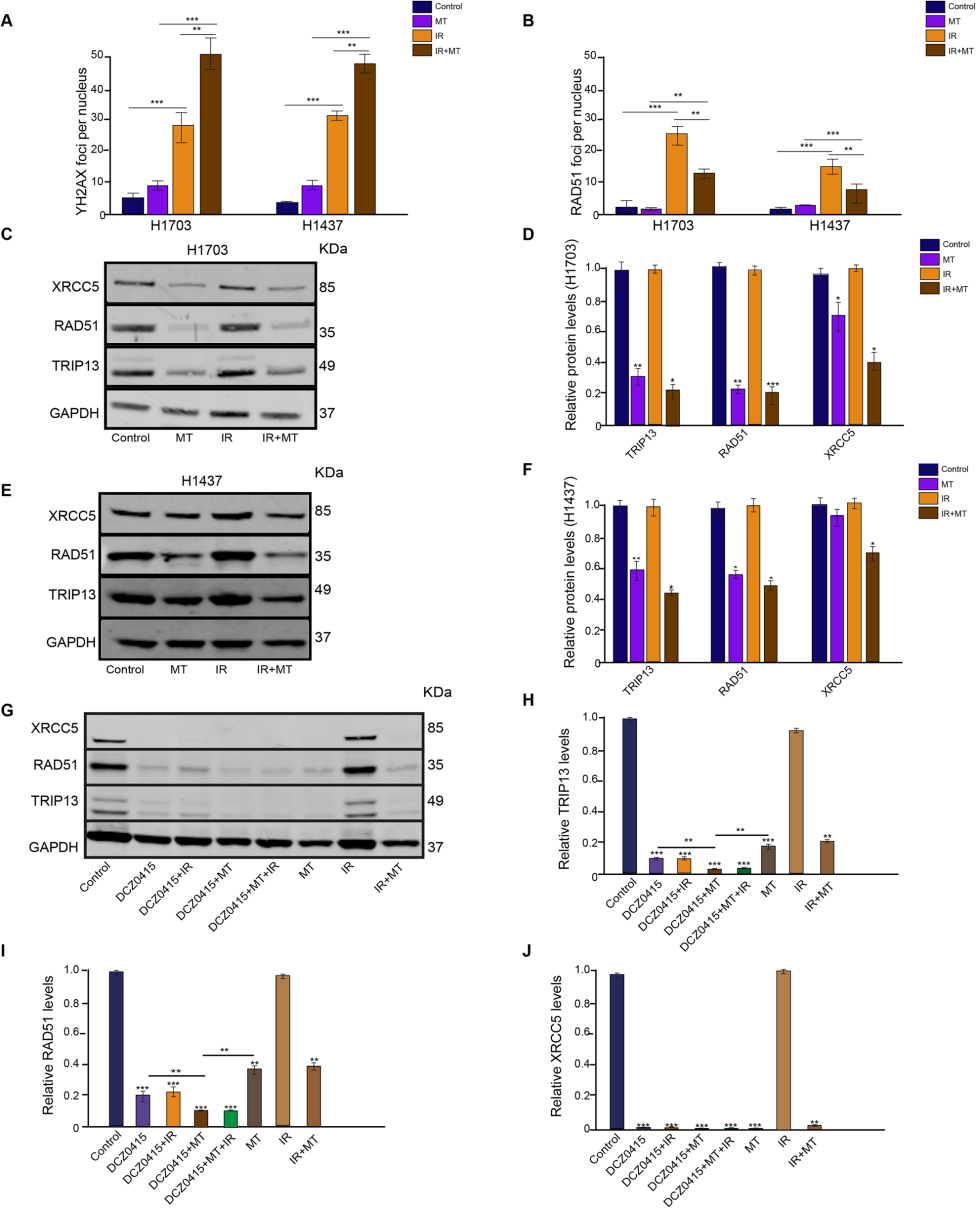
Melatonin enhances IR sensitivity through suppression TRIP13. **A** number of γ-H2AX foci per nucleus of control, MT, IR, and IR with MT treated H1703 and H1437 cells. **B** number of RAD51 foci per nucleus of control, MT, IR, and IR with MT treated H1703 and H1437 cells. **C** Expression of XRCC5, RAD51, and TRIP13 levels of control, MT, IR, and IR with MT treated H1703 cells. **D** XRCC5, RAD51, and TRIP13 protein levels quantified relative to GAPDH of control, MT, IR, and MT + IR treated H1703 cells. **E** Expression of XRCC5, RAD51, and TRIP13 levels of control, MT, IR, and IR with MT treated H1437cells. **F** XRCC5, RAD51, and TRIP13 protein levels quantified relative to GAPDH of control, MT, IR, and MT + IR treated H1437 cells. **G** Expression of XRCC5, RAD51, and TRIP13 levels of control, DCZ0415, DCZ0415 + IR, DCZ0415 + MT, DCZ0415 + MT + IR, MT, IR and IR with MT treated H1703 cells. **H** TRIP13 protein level quantified relative to GAPDH of control, DCZ0415, DCZ0415 + IR, DCZ0415 + MT, DCZ0415 + MT + IR, MT, IR, and IR with MT treated H1703 cells. **I** RAD51 protein level quantified the relative to GAPDH in control, DCZ0415, DCZ0415 + IR, DCZ0415 + MT, DCZ0415 + MT + IR, MT, IR, and IR with MT treated H1703 cells. **J** XRCC5 protein level quantified relative to GAPDH of control, DCZ0415, DCZ0415 + IR, DCZ0415 + MT, DCZ0415 + MT + IR, MT, IR, and IR with MT treated H1703 cells. *n* = 3, *p < 0.05. **p < 0.01. ***p < 0.001.

Previously, we discovered that TRIP13 promotes HR and NHEJ after IR treatment of NSCLC cells [25]. To confirm whether MT induced downregulation of TRIP13 also affects radiation-induced DNA damage and repair, we treated cells with MT in combination with IR. First, we performed immunofluorescent staining of γ-H2AX and RAD51 (Supplementary Fig. S4A, B). In combination with MT, the number of IR-induced γ-H2AX foci was significantly higher in both cell lines (Fig. 6A). In line with this observed increase in remaining DSBs, the number of IR-induced RAD51 foci, indicating HR-mediated repair of these DSBs, was significantly lower when MT was added (Fig. 6B). These results suggest that, in NSCLC cells, treatment with MT reduces HR-mediated DNA repair (fewer RAD51 foci), which causes more remaining DSBs (more γ-H2AX foci).

To further investigate how MT downregulates TRIP13 and IR-induced DNA repair, we performed Western blot analyses for the proteins XRCC5 (NHEJ), RAD51 (HR) and TRIP13. MT, with and without IR, again significantly decreased TRIP13 protein expression (Fig. 6C–F). Protein levels of RAD51and XRCC5 were decreased in both cell lines in response to MT (Fig. 6C–F). These results show that MT inhibits IR-induced HR and NHEJ in both cell lines.

To investigate whether these effects of MT were mediated via TRIP13, we used a TRIP13 knockout cell line to detect relative protein expression levels. However, the levels of XRCC5 and RAD51 were too low/absent in the H1703-TRIP13KO cell line (Supplementary Fig. 5A, B). The TRIP13KO cells displayed very low TRIP13 expression, most likely due to occasional ribosomal frameshifting or transcriptional slippage resulting in restoration of the original reading frame [27, 28].

To circumvent these low expression values in the TRIP13KO cells, we instead used the TRIP13 inhibitor DCZ0415 and H1703 cells. Whether MT has a direct effect on RAD51 and XRCC5, or if it primarily acts through TRIP13, was determined using Western blot analysis to assess the effects of DCZ0415 on MT and TRIP13, RAD51, and XRCC5 expression (Fig. 6G). The results indicated that DCZ0415 alone significantly reduced both TRIP13 and RAD51, with approximately an 85% reduction in TRIP13 and an 80% reduction in RAD51. Similarly, MT alone also decreased TRIP13 (80%) and RAD51 (60%). When cells were treated with both DCZ0415 and MT, we observed a decrease in TRIP13 (95%) and RAD51 (90%) (Fig. 6H, I). These results indicate that MT has a partial direct effect on RAD51. However, the stronger suppression of RAD51 when both DCZ0415 and MT are used together suggests that MT’s effect on RAD51 is primarily mediated through TRIP13 inhibition.

Since treatment with DCZ0415 or MT resulted in no detectable XRCC5 expression, it was impossible to determine from these results whether MT has a direct effect on XRCC5 or whether it acts through TRIP13 (Fig. 6J).

### Increase in the anti-proliferative effects of melatonin in combination with DCZ0415

**Fig. 7:**
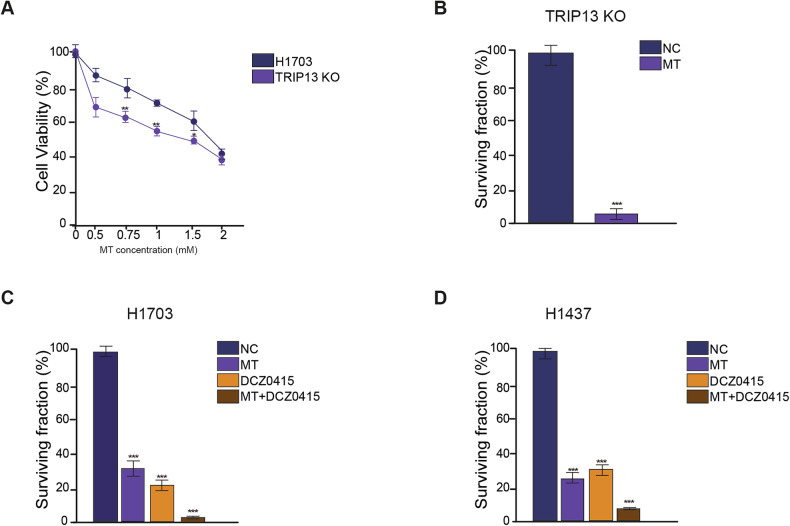
Additive improvement of anti-proliferative effects on NSCLC cells by melatonin in combination with DCZ0415. **A** Percentage of cell viability of H1703 and TRIP13-KO cells using Alamar Blue. **B** percentage of the clonogenic survival in TRIP13-KO cells with or without 1 mM MT. **C** Percentage of the clonogenic survival of control, MT, DCZ0415, and MT + DCZ0415 treated H1703 cells. **D** Percentage of the clonogenic survival of control, MT, DCZ0415, and MT + DCZ0415-treated H1437 cells. *N* = 3, *p < 0.05. **p < 0.01. **p < 0.001.

To confirm whether the effects of MT are mediated via TRIP13, we used the TRIP13-KO cells (H1703) we generated previously [14]. We performed a cell viability assay to detect the effect of different concentrations of MT on H1703 and TRIP13-KO cells. The results showed that after treatment with varying concentrations of MT, cell viability significantly decreased in TRIP13-KO cells compared to H1703 cells (Fig. 7A). Additionally, treatment of TRIP13-KO cells with 1 mM MT resulted in a decrease in the surviving fraction compared to untreated cells as measured by a clonogenic assay (Fig. 7B, Supplementary Fig. 5C).

Given the high TRIP13 expression in H1703 and H1437 cells, we tested the effects of the TRIP13 inhibitor DCZ0415 and MT. We subjected H1703 and H1437 cells to four treatments: Control, Melatonin, DCZ0415, and a combination of Melatonin and DCZ0415. Clonogenic assays demonstrated that the surviving fractions of both cell lines treated for ~10 days further decreased following TRIP13 downregulation in combination with treatment with DCZ0415 and Melatonin (Fig. 7C, D, Supplementary Fig. 5D, E). Taken together, the combination of melatonin and the TRIP13 inhibitor resulted in enhanced anti-proliferative effect on NSCLC cells (Fig. 7C, D).

## Discussion

Our previous research identified that TRIP13 plays a key role in the development of resistance to IR. In patients with NSCLC, TRIP13 expression appears to be correlated with a poor prognosis [25]. Here we find that melatonin (MT) not only downregulates TRIP13 but also the DNA repair proteins RAD51 and XRCC5, thereby inhibiting aberrant DNA repair in cancer cells. Since treatment with TRIP13 inhibitor DCZ0415 or MT resulted in no detectable XRCC5 expression, it is difficult to determine whether MT has a direct effect on XRCC5 or if it primarily acts through TRIP13 (Fig. 5J). However, our previous research has shown that both XRCC5 and RAD51 are downstream of TRIP13 and that knockout of TRIP13 significantly decreases the expression of both XRCC5 and RAD51 proteins [25]. Therefore, it seems that MT’s effect on both RAD51 and XRCC5 is primarily mediated through TRIP13 inhibition. Additionally, we discovered that MT acts through MT receptor MTNR1B rather than MTNR1A.

MT, mostly known as a hormone secreted by the pineal gland and involved in the circadian rhythm, has gained attention for its anticancer properties in various malignancies [29]. MT inhibits tumor processes in various ways, including by reducing oxidative stress [30], inhibiting proliferation and metastasis [31], promoting apoptosis [32], activating the immune system, and causing metabolic disorders [33]. MT has strong inhibitory effects on NSCLC and inhibits proteins, such as PD-L1, CDK4, and TOX3, thereby hindering proliferation and promoting apoptosis of NSCLC cells [5, 34, 35]. Our data further confirm the strong inhibitory effects of MT on NSCLC cells, consistent with previous studies [5, 7, 33]. In line with the established mechanisms by which MT suppresses cell growth and migration [6, 36], we identified a novel pathway in which MT modulates TRIP13, resulting in a significant reduction in cell survival. This discovery expands the current understanding of MT’s anticancer effect. adding TRIP13 as a critical target that contributes to MT’s anticancer properties. Moreover, while MT typically regulates survival signaling and tumor progression through its G protein-coupled membrane receptors, MTNR1A and MTNR1B [4], our findings suggest a receptor-specific effect. Specifically, we observed that MT exerts its inhibitory effect primarily through MTNR1B, rather than MTNR1A. This discovery refines our understanding of MT’s receptor-mediated actions and offers new insight into its selective targeting potential in NSCLC therapy. By identifying both TRIP13 modulation and MTNR1B-specific inhibition, our study contributes novel data to the growing body of research on MT’s therapeutic role in LUAD.

The rising global incidence of lung cancer has elevated it to the status of the most common malignant tumor [36]. Surgery and chemo/radiotherapy persist as the established norms for clinical therapy in lung cancer [37]. In this study, we tested the combination of MT with IR in NSCLC cells (Fig. 6A, B, and Supplementary Fig. 5A, B). To assess the effects of this combination on DNA damage and repair, we performed staining for γ-H2AX and RAD51. γ-H2AX foci were used as a marker for DNA double-strand breaks (DSBs), while RAD51 foci were assessed to evaluate HR repair. Our results showed that γ-H2AX foci were significantly increased in cells treated with both MT and IR, indicating more DNA double-strand breaks (DSBs). In contrast, RAD51 foci formation was reduced in the same cells, suggesting that MT interferes with the HR-mediated repair of DNA DSBs. These findings highlight that MT can inhibit DNA repair mechanisms, leading to the accumulation of DNA damage and potentially enhancing the effectiveness of IR in treating NSCLC.

Recently, targeted gene-specific therapies have become more popular, particularly in the field of cancer. Many oncogenes contribute to the acquisition of cancer-specific hallmarks that play a crucial role in the transformation of healthy cells into cancerous ones [8]. These oncogenes contribute to the development of distinctive characteristics associated with cancer, including unrestricted cell proliferation, genomic instability, and irregular apoptosis regulation. Many contemporary cancer therapies effectively target these fundamental processes [38]. By comparing the transcriptomes of dissected germ cell subtypes with the publicly accessible transcriptomes of different healthy somatic tissues and tumor samples, we previously found that many of these oncogenes are normally restricted to the germline, the so-called germ cell cancer genes (GC genes) [9, 10]. Targeting GC-genes is anticipated to result in fewer side effects, perhaps even only temporary infertility. Our previous research reported that NSCLC cells that express many GC genes indeed are more proliferative and resistant to radiation-induced DNA damage [13]. Importantly, this phenotype could be reduced by inhibition or knockout of TRIP13 [13].

In our study, we observed that TRIP13 knockout (KO) cells exhibited increased sensitivity to MT treatment, which may be attributable to several potential mechanisms. First, TRIP13 is involved in the regulation of DNA damage repair [25]. In its absence, cells might therefore experience defects in the repair of DNA damage, leading to an accumulation of cellular stress. This may make them more vulnerable to MT’s cytotoxic effects. Second, TRIP13 is involved in regulating various cellular processes, including mitotic progression and cell proliferation regulation [25], and is part of the mitotic spindle checkpoint, which ensures proper chromosome segregation during cell division [39]. Without TRIP13, the cell cycle might thus become dysregulated, potentially leading to abnormal mitosis and increased genomic instability. Since MT is known to have a variety of cellular effects, including modulation of cell cycle progression and apoptosis [40], loss of TRIP13 may lead to an altered cellular environment that is more sensitive to these effects. Hence, MT’s impact on cellular proliferation could be more pronounced in TRIP13 KO cells because these cells might have an already destabilized genomic state, making them more susceptible to the effects of MT.

In the current study, we found that MT treatment also downregulates the expression of TRIP13. Hence, combined treatment with MT and the TRIP13 inhibitor DZ0415 may lead to improved outcomes for NSCLC patients, while side effects may be only apparent in the male gonad due to the role of TRIP13 in meiosis [13]. A mouse model for NSCLC could be utilized to test this hypothesis. However, the physiology, cellular biology, and (epi)genetics of mouse NSCLC may differ too significantly from those of human NSCLC to draw reliable conclusions regarding suitable treatments [41–43]. Moreover, these fundamental differences raise ethical concerns about subjecting mice to potentially painful or harmful treatments when the translational relevance to human disease remains uncertain in this case.

While it is true that DCZ0415 is not yet a clinically approved agent, it has consistently demonstrated both in vitro and in vivo efficacy and specificity in cancer, as shown in our studies [1, 2] and other previous publications [44–46]. This consistent performance validates its role as a reliable research tool for investigating TRIP13 function. Although it is not yet suitable for immediate clinical application, these promising results suggest that DCZ0415 may hold potential for future use in therapies, once further development, including pharmacokinetic and toxicity studies, is completed. Therefore, in combination with knock-out/down experiments to check specificity (this study and [13]), its use in our study provides valuable insights into TRIP13 inhibition and its implications for cancer treatment, laying the groundwork for future therapeutic strategies.

Additionally, our results revealed that the expression of TRIP13 was higher in LUAD and LUSC tissues than in normal lung tissues and that higher TRIP13 expression correlates with LUAD patients’ poor prognoses. Moreover, elevated TRIP13 expression was significantly correlated with increasing stages of LUAD and LUSC, indicating its potential role as a biomarker for NSCLC progression.

Our study provides evidence that TRIP13 expression plays a role in modulating cellular sensitivity to MT. However, we also observed that the relationship between TRIP13 and MT response is more complex than initially anticipated. Specifically, our data show that MT treatment leads to a downregulation of TRIP13 expression (Fig. 3), and that TRIP13 knockout cells exhibit even stronger sensitivity to MT than their wild-type counterparts (Fig. 7). These findings imply that MT does not simply act through TRIP13 but rather that MT may suppress TRIP13 expression as part of its mechanism. This observation leads us to propose that TRIP13 downregulation is likely a consequence of MT treatment, rather than being a driver of MT’s cytotoxic effects. The increased sensitivity of TRIP13 knockout cells may reflect a loss of protective mechanisms that are normally conferred by TRIP13, such as its role in mitotic regulation [41] or maintenance of genome stability during stress conditions [47]. Interestingly, while TRIP13 appears to have a protective function against MT toxicity (Fig. 7A), it is also possible that MT affects additional cellular pathways beyond TRIP13, contributing to the overall sensitivity observed. Hence, these results open the door to exploring additional signaling mechanisms that may modulate MT sensitivity. Future studies should investigate the downstream effects of TRIP13 loss and the broader signaling networks involved in MT response. Specifically, it will be important to assess the role of spindle assembly checkpoint proteins, DNA repair mechanisms, and stress response pathways in mediating MT sensitivity, as these could provide valuable insights into how MT interacts with the cellular machinery beyond TRIP13.

Our findings highlight the potential of melatonin as a therapeutic agent in NSCLC by downregulating TRIP13, especially when used in combination with the TRIP13 inhibitor DCZ0415. Given the role of TRIP13 in LUAD and LUSC stage progression, and its function in DNA double-strand break repair, multi-targeting this protein could offer an improved therapeutic strategy for LUAD and LUSC patients with minimal side effects.

## Materials and methods

### Experimental model and subject details

Cells (H1703 (ATCC, LUSC), *TRIP13* KO in H1703 [13] and H1437 (ATCC, LUAD)) were selected based on expression of *TRIP13* and germline cancer (GC) genes, culture compatibility, or cancer origin and characteristics as described in previous research [13], in which also the pathology type and mutational background of these cells are described. The cells were maintained in 5% CO2 at 37°C in RPMI-1640 medium (Thermo Fisher Scientific), supplemented with 10% fetal bovine serum (Thermo Fisher Scientific), 1% HEPES (Gibco), 1% Pen-Strep (Gibco), and 2.2% glucose (Gibco). The cells were refreshed every 3 days and routinely passaged for use. Throughout the entire study, the cells in culture tested negative for mycoplasma contamination. MT treatment for 48 h began after the cells were adherent, and the medium was changed every day to ensure the effectiveness of MT.

### Irradiation

The cell lines were subjected to 2 Gy of ionizing radiation (IR) in the CellRad system (OMIYS, Netherlands), based on previous results obtained for H1703 and H1437 cells [13].

### Clonogenic assays

Clonogenic assays were performed as described previously [13]. Briefly, cells in both the no-treatment and treatment groups were plated at an equal density in triplicate in six-well plates. 4 hours after plating, cells were exposed to 2 Gy of IR. Once the negative control condition (i.e., 0 mM MT) showed the formation of colonies of 50 cells, which took ~14 days. The medium was removed, and cells were gently washed with phosphate-buffered saline (PBS). Subsequently, the cells were fixed and stained with 6% glutaraldehyde + 0.5% crystal violet in PBS. The numbers of colonies with more than 50 cells were electronically counted with Image J (version1.54i) and manually confirmed.

### Cell viability assay

Cells were seeded in triplicate in 96-well plates. At ~70–80%, the cells were treated with different concentrations MT (0 mM, 0.5 mM, 0.75 mM, 1 mM, 1.5 mM, 2 mM). Cell viability was measured using the Alamar Blue assay (BUF012B, BIO-RAD) according to the manufacturer’s protocol.

### Cell scratch (wound healing) assay

Cells were cultured in Incucyte ® image lock 96-well-plates (Satorius BA-04855). When the cells reached around 90% confluency, the Incucyte 96-well wound marker tool (Sartorius 4563) was used following the protocol provided by the manufacturer. After a scratch was automatically set, the cells were gently washed to remove detached cells, and the medium was refreshed with medium with or without MT. Wound healing was assessed using the Incucyte Live Cell Analysis System and quantified using Image J (version 2.0).

### Proliferation assay

Cells were seeded in 96-well plates. After cell adherence, cells were exposed to IR in a CellRad system and/or 1 mM MT. When cells reached 50–60% confluency, the 5-ethynyl-2’-deoxyuridine (EdU) was added to the culture for 2 hours. Quantification of EdU-positive cells was performed using the Cell Proliferation Kit (C10337, Thermo Fisher Scientific) as previously described [20] and represented by the mean ± SEM of three independent experiments. Images were analyzed using Leica Application Suite X, and counting of nuclei and EdU stains was performed electronically in Image J (version1.54i).

### Western blotting (for full-length western blots, see Supplemental Material)

Cells were seeded in six-well plates. After treating cells for 48 h with MT, the media was discarded and cells were washed twice with PBS. Cells were treated with 1 mL 0.25% trypsin in the incubator and, after 5 min, RPMI 1640 (with 10% FCS, 1% Hepes, 1% Penstraps, 1.1% glucose) was added to block the trypsin activity. The cells were pipetted from the plates to tubes. Proteins were extracted from the cells using a RIPA buffer with the addition of protease (cOmplete™ Protease Inhibitor Cocktail, Roche) and phosphatase inhibitors (PhoSTOP™, Roche). Protein expression was quantified with the Qubit Protein Assay Kit (Thermo Fisher Scientific). Western blot analysis was performed using the LI-COR Odyssey imaging system (LI-COR Biosciences) as previously reported [19]. The primary antibodies used were against TRIP13 (1:1000, ab128171, Abcam), GAPDH (1:1000; FL-335, Santa Cruz Biotechnology), mouse anti-TUBULIN (1:1000; T9026, Sigma), PCNA (1:1000, ab92552, Abcam), MTNR1A (1:1000, sc390328, Santa Cruz Biotechnology), MTNR1B (1:1000, ab203346, Abcam), RAD51 (1:1000; PA5-27195, Thermo Fisher Scientific), XRCC5 (1:1000, ab2172-500, Abcam). Secondary antibodies were IRDye 800CW Goat Anti-Rabbit IgG (H + L) (1:1000, 926-32211, LiCor), IRDye 800CW Goat Anti-Mouse IgG (H + L) (1:1000, 926-32210, LiCor). GAPDH was used as a loading control on the same blot, or the same volumes were loaded from the same experimental sample. Band intensities were assessed using Image Studio Lite (Version 5.5).

### γ-H2AX and RAD51 staining

Cells were cultured on multi Nunc Lab-Tek II chambered slides (Thermo Fisher Scientific) for MT treatment 48 hours, after which they were exposed to 2 Gy of irradiation in a CellRad system (Precision X-Ray). Cells were fixated at three hours after irradiation in 4% paraformaldehyde for 10 min and subsequently permeabilized in PBS with 0.1% triton-X for 15 min. Non-specific adhesion sites were blocked for 45 minutes in 0.25% Tween-20/PBS with 1% bovine serum albumin, followed by the addition of primary antibodies against γ-H2AX (1:10.000, 05-636, Millipore) or RAD51 (1:50, PA5-27195, Thermo Fisher Scientific), or isotype immunoglobulin G in the case of negative controls. After overnight incubation at 4°C, the cells were washed and incubated with the corresponding host-specific secondary antibodies goat anti-mouse Alexa Fluor 488 (1:1.000, A11029, Thermo Fisher Scientific) or goat anti-Rabbit Alexa Fluor 532 (1:1000, A11009, Life Technologies), and counterstained with DAPI. The slides were mounted with Prolong Gold anti-fade (Thermo Fisher Scientific) and visualized using a Leica DM5000B microscope. γ-H2AX and RAD51 foci within the cell nucleus were counted manually in at least 100 cells per condition. This was repeated three times for statistical analysis.

### Quantitative real-time PCR (Q-PCR)

Total RNA was extracted from control and treated cells using the RNeasy Mini Kit (Qiagen) and following the protocol provided by the manufacturer. The RNA samples were reverse transcribed using the SensiFAST cDNA Synthesis Kit (Bioline). The synthesized cDNA was then used for Q-PCR reactions, using the Roche LightCycler 480 platform in a 384-well plate format. The Q-PCR reaction was performed in a 10 µL. volume system including 2X LightCycler 480 SYBR Green I Master (Roche). *GAPDH*, *ACTB*, and *TUBA1C* were used as reference genes. The data were analyzed using the ΔΔCt method. The primers for Q-PCR analysis are listed in Table 1.

**Table 1:** Primer sequences for Q-PCR analysis.

Gene	Forward primer	Reverse primer	Gene
*Gapdh*	GTCTCCTCTGACTTCAACAGCG	ACCACCCTGTTGCTGTAGCCAA	*Gapdh*
*Actb*	ACCAGAGGCATACAGGGAC	CTAAGGCCAACCGTCAAAAG	*Actb*
*Tuba1c*	AGCGTGCCTTTGTTCACT	CTCATCCTCTCCGTCAGC	*Tuba1c*
*Trip13*	CGGGTCCTGAGAAAACTCCC	CAAACTGCTTGTCCACTGCC	*Trip13*

### Generation of siMT1 or siMT2 in H1703 cells

H1703 cells were transfected with MEL-1A-R siRNA plasmid (sc-35917; Santa Cruz Biotechnology), MEL-1B-R siRNA plasmid (sc-40114; Santa Cruz Biotechnology), or Control siRNA plasmid (sc-37007; Santa Cruz Biotechnology) using X-tremeGENE™ HP DNA Transfection Reagent (06366236001; Sigma) following the protocol provided by the manufacturer. After 48 h, cell pellets were collected for Western blot analysis.

### Statistical analysis

The data obtained were analyzed in Prism Graph Pad. A Shapiro–Wilk test was used to validate the normal distribution of the data. For comparison of two groups, two-sided *T* tests were performed. For comparisons involving more than two groups, one-way ANOVA was performed. Results were corrected for multiple testing using the Bonferroni correction where appropriate. p < 0.05 was regarded as statistically significant.

## Supplementary information

SUPPLEMENTAL MATERIAL

Full-length western blots

## Data Availability

The data that support the findings of this study are available from the corresponding author upon reasonable request.
