# Supplementary material for: Melatonin suppresses cancer cell proliferation, DNA repair and expression of the oncogene TRIP13
Source: Cell Death Discov. 2025 Oct 27;11:489. doi: 10.1038/s41420-025-02788-z (PMC12559315; doi:10.1038/s41420-025-02788-z)
Supplement: Supplementary file 1 — SUPPLEMENTAL MATERIAL [file 41420_2025_2788_MOESM1_ESM.pdf]

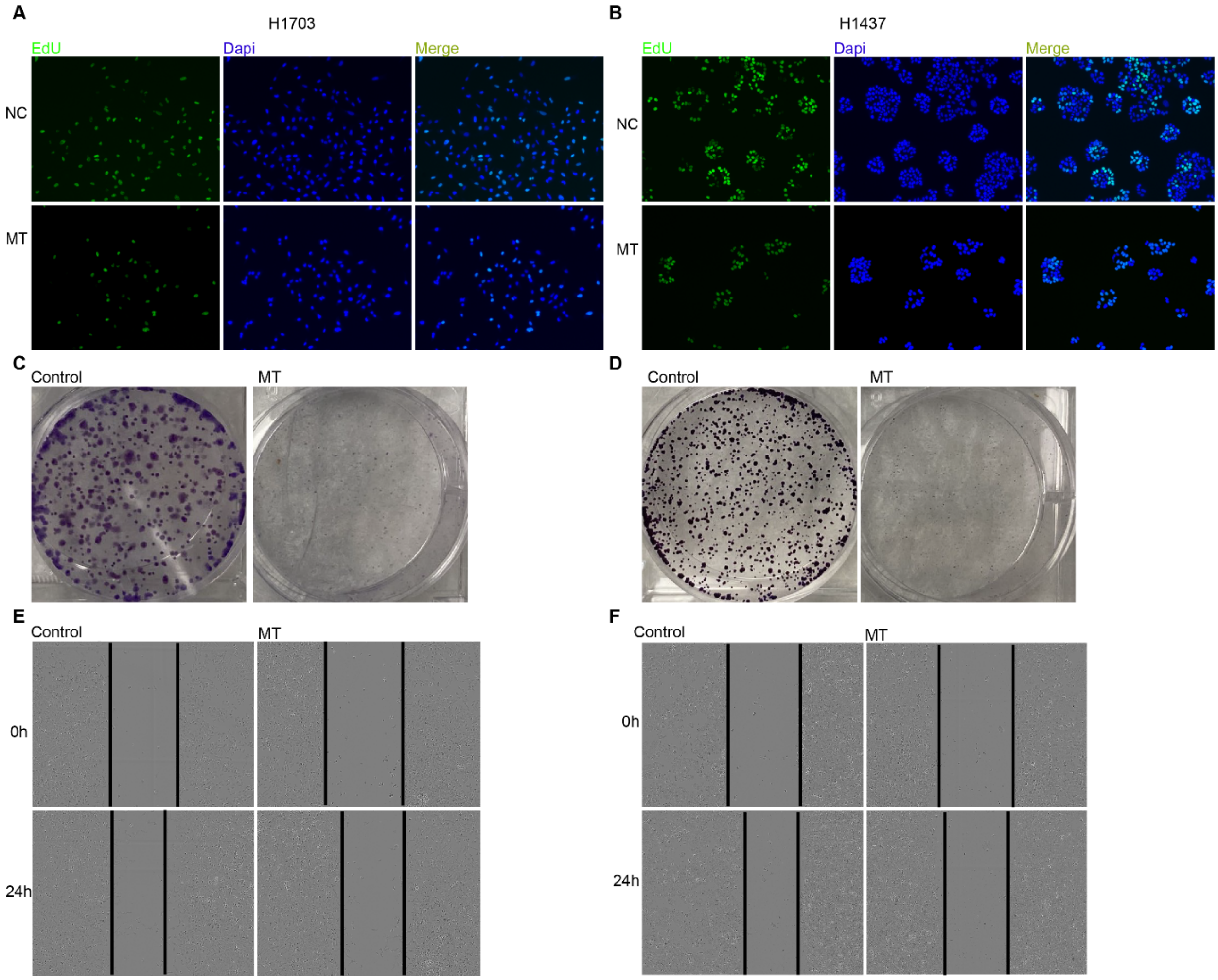

Figure S1*. *Melatonin (MT) inhibits LUAD and LUSC cell proliferation and wound healing.

Representative images of ***(A)*** *H1703 (LUSC) cells and* ***(B)*** *H1437 (LUAD) cells with or without 1mM MT treatment that incorporated 5-ethynyl-2'-deoxyuridine (EdU) (green). Cells were counterstained with DAPI (blue) to visualize nuclei (n=9). Representative images of* ***(C)*** *H1703 and* ***(D)*** *H1437 cells with or without 1mM MT treatment from clonogenic survival assays (n=3). Representative images of* ***(E)*** *H1703 and* ***(F)*** *H1437 cells treated with and without 1mM MT (x10 magnification) from Wound healing assays.*

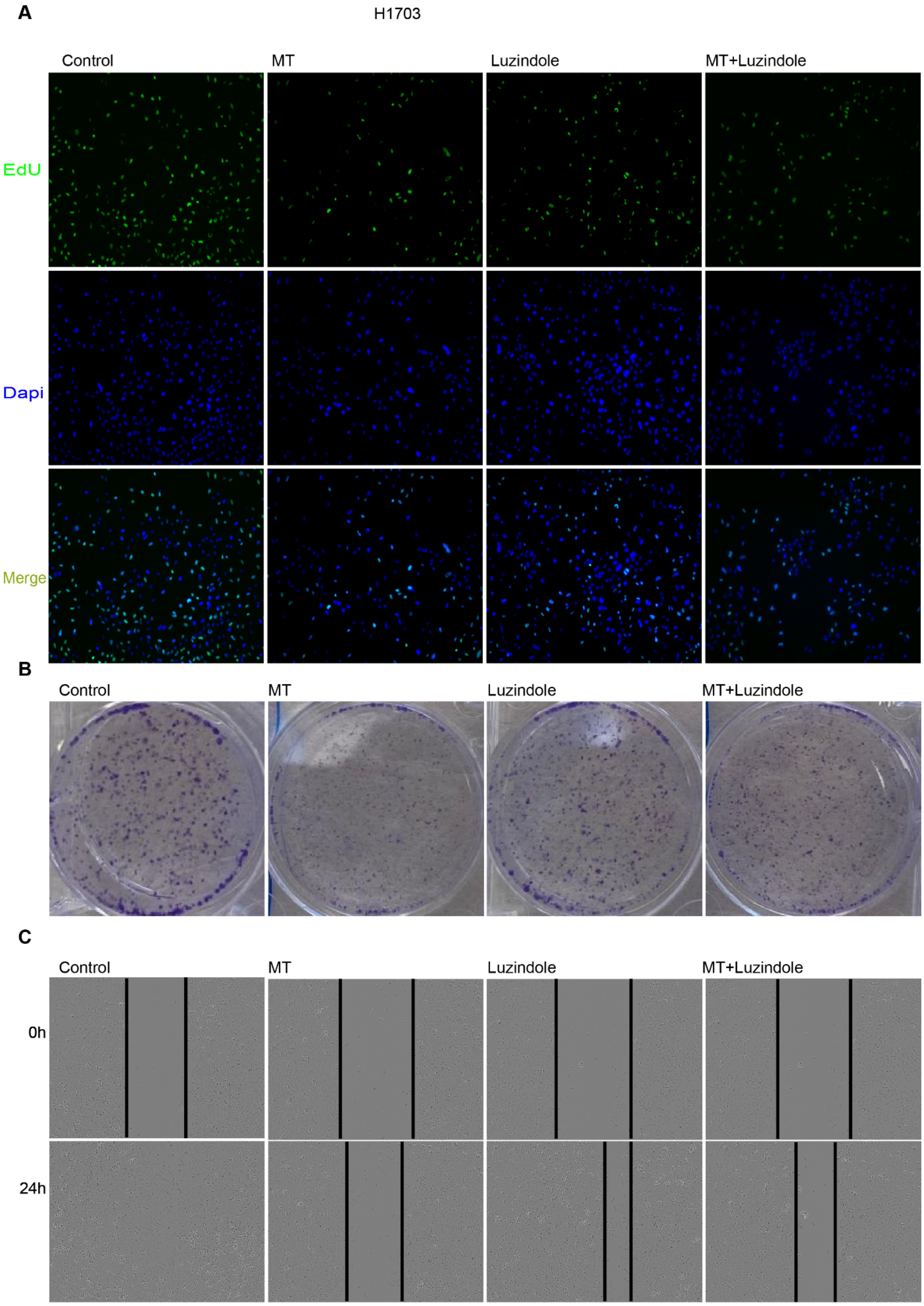
A
H1703
Control
MT
Luzindole
MT+Luzindole
EdU
Dapi
Merge
B
Control
MT
Luzindole
MT+Luzindole
C
Control
MT
Luzindole
MT+Luzindole
0h
24h

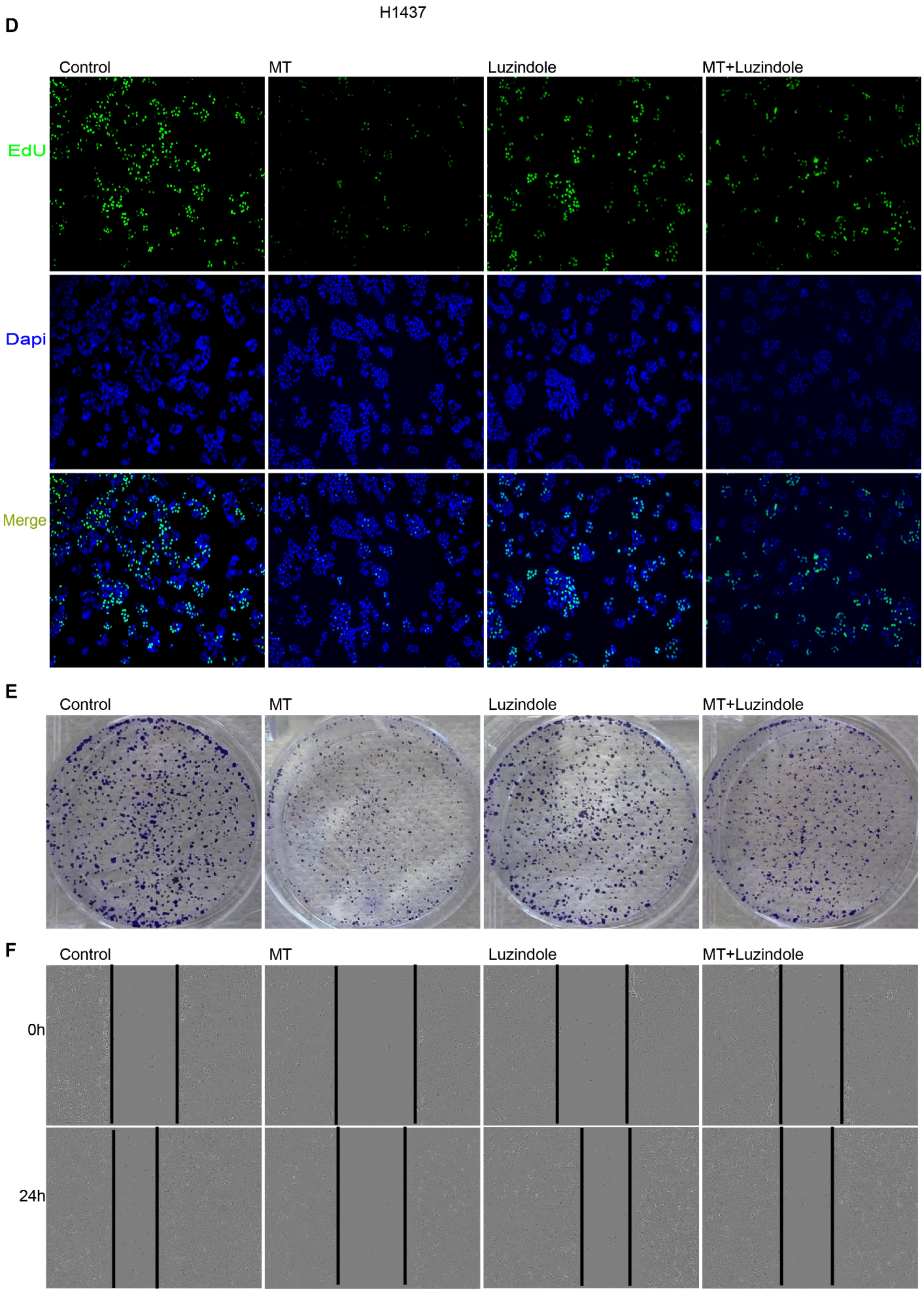

H1437
D
Control
MT
Luzindole
MT+Luzindole
EdU
Dapi
Merge
E
Control
MT
Luzindole
MT+Luzindole
F
Control
MT
Luzindole
MT+Luzindole
0h
24h

Figure S2. Blockage of MTNR1B impairs melatonin's ability to reduce cell proliferation.

Representative images of **(A)** H1703 cells and **(D)** H1437 cells treated with control, MT, Luzindole and MT+Luzindole that incorporated 5-ethynyl-2'-deoxyuridine (EdU) (green). Cells were counterstained with DAPI (blue) to visualize nuclei. Representative images of **(B)** H1703 cells and **(E)** H1437 cells treated with control, MT, Luzindole and MT+Luzindole from clonogenic survival assays. Representative images of **(C)** H1703 cells and **(F)** H1437 cells treated with control, MT, Luzindole and MT+Luzindole cells from Wound healing assays.

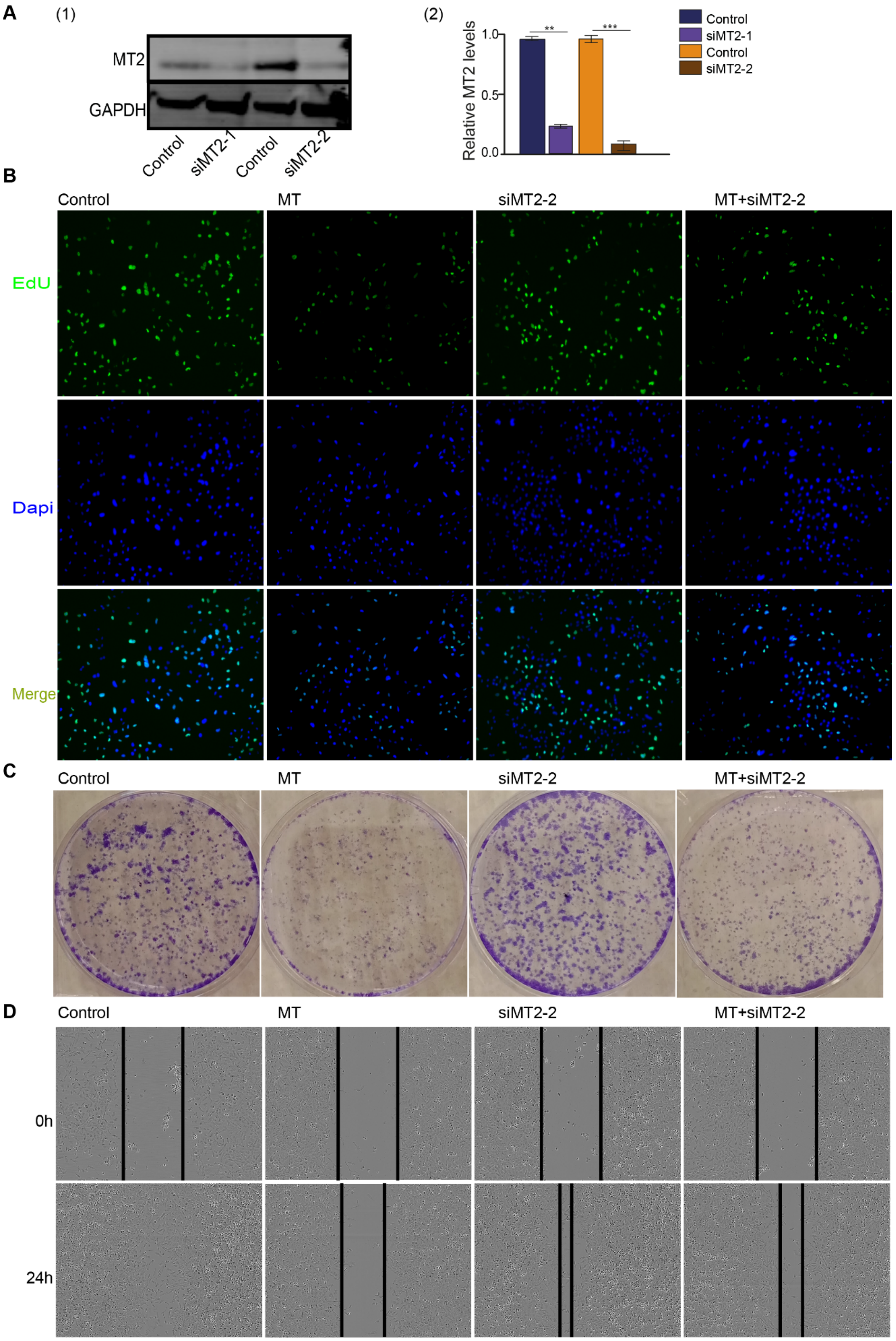
A
(1)
MT2
GAPDH
Control
siMT2-1
Control
siMT2-2
(2)
Relative MT2 levels
1.0
0.5
0.0
**
***
Control
siMT2-1
Control
siMT2-2
B
Control
MT
siMT2-2
MT+siMT2-2
EdU
Dapi
Merge
C
Control
MT
siMT2-2
MT+siMT2-2
D
Control
MT
siMT2-2
MT+siMT2-2
0h
24h

Figure S3. Knockdown of MT2 impairs melatonin's ability to reduce cell proliferation.

(A) *(1) Expression of MT2 protein in control and siMT2-1 and, control and siMT2-2 treated in the H1703 cells. (2) MT2 protein levels quantified relative to GAPDH in control and siMT2-1, and control and siMT2-2 treated H1703 cells (n=3).* ***(B)*** *Representative images of H1703 cells treated with control, MT, siMT2-2 and MT+siMT2-2 that incorporated 5-ethynyl-2'-deoxyuridine (EdU) (green). Cells were counterstained with DAPI (blue) to visualize nuclei.* ***(C)*** *Representative images of clonogenic survival assays* ***of*** *H1703 cells treated with control, MT, siMT2-2, or MT+siM2-2 from.* ***(D)*** *Representative images of wound healing assays using H1703 cells treated with control, MT, siMT2-2, or MT+siMT2-2.*

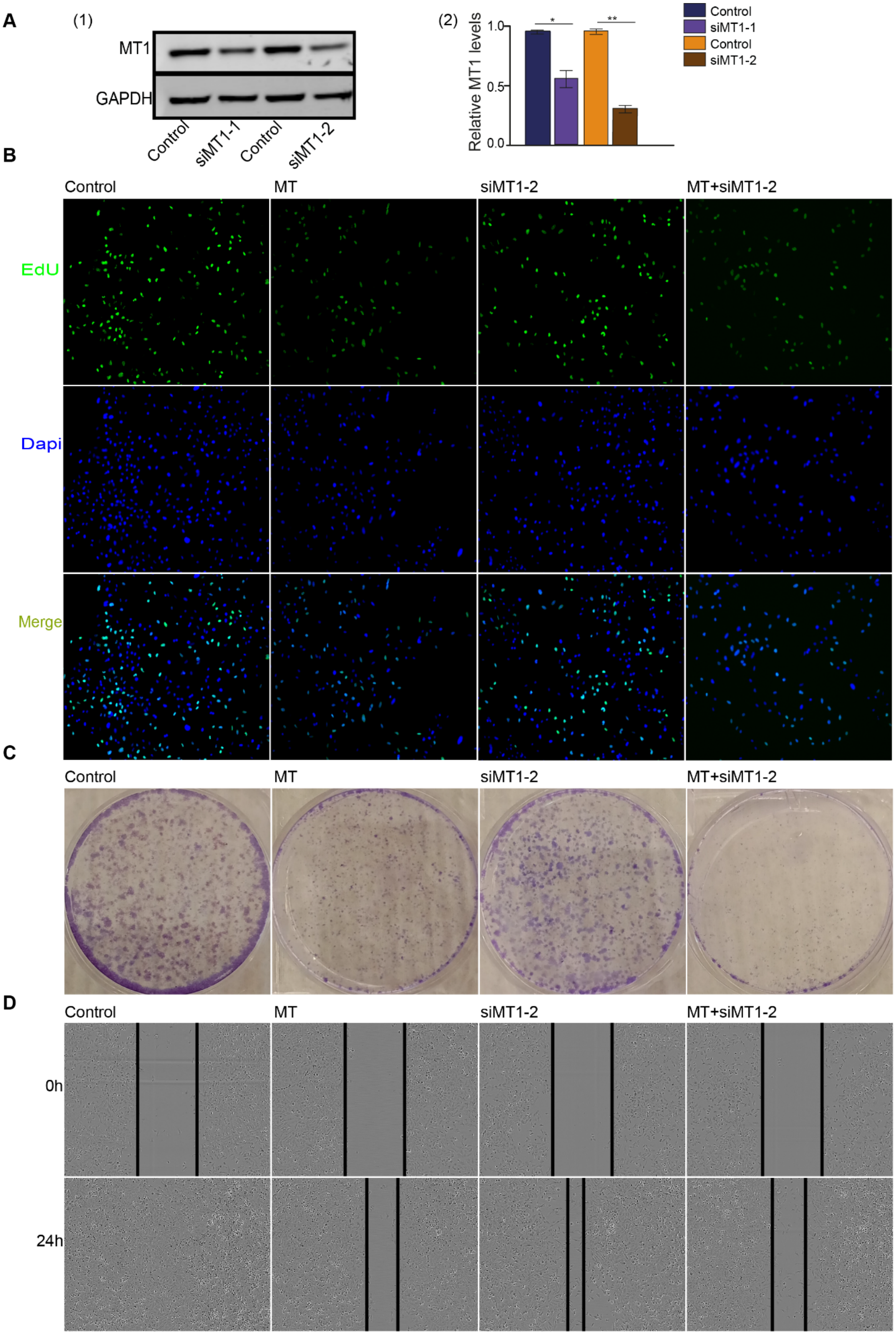
A
(1)
MT1
GAPDH
Control
siMT1-1
Control
siMT1-2
(2)
Relative MT1 levels
1.0
0.5
0.0
*
**
Control
siMT1-1
Control
siMT1-2
B
Control
MT
siMT1-2
MT+siMT1-2
EdU
Dapi
Merge
C
Control
MT
siMT1-2
MT+siMT1-2
D
Control
MT
siMT1-2
MT+siMT1-2
0h
24h

Figure S4. Knockdown of MT1 does not impair melatonin's ability to reduce cell proliferation

(A) (1) Expression of MT1 protein in control and siMT1-1, and control and siMT1-2 treated in the H1703 cells. (2) MT1 protein levels quantified relative to GAPDH in contro and, siMT1-1, and control and siMT1-2 treated H1703 cells (n=3) (B) Representative images of H1703 cells treated with control, MT, siMT1-2 and MT+siMT1-2 that incorporated 5-ethynyl-2'-deoxyuridine (EdU) (green). Cells were counterstained with DAPI (blue) to visualize nuclei. (C) Representative images of clonogenic survival assays of H1703 cells treated with control, MT, siMT1-2, or MT+siMT1-2 from. (D) Representative images of wound healing assays using H1703 cells treated with control, MT, siMT1-2, or MT+siMT1-2.

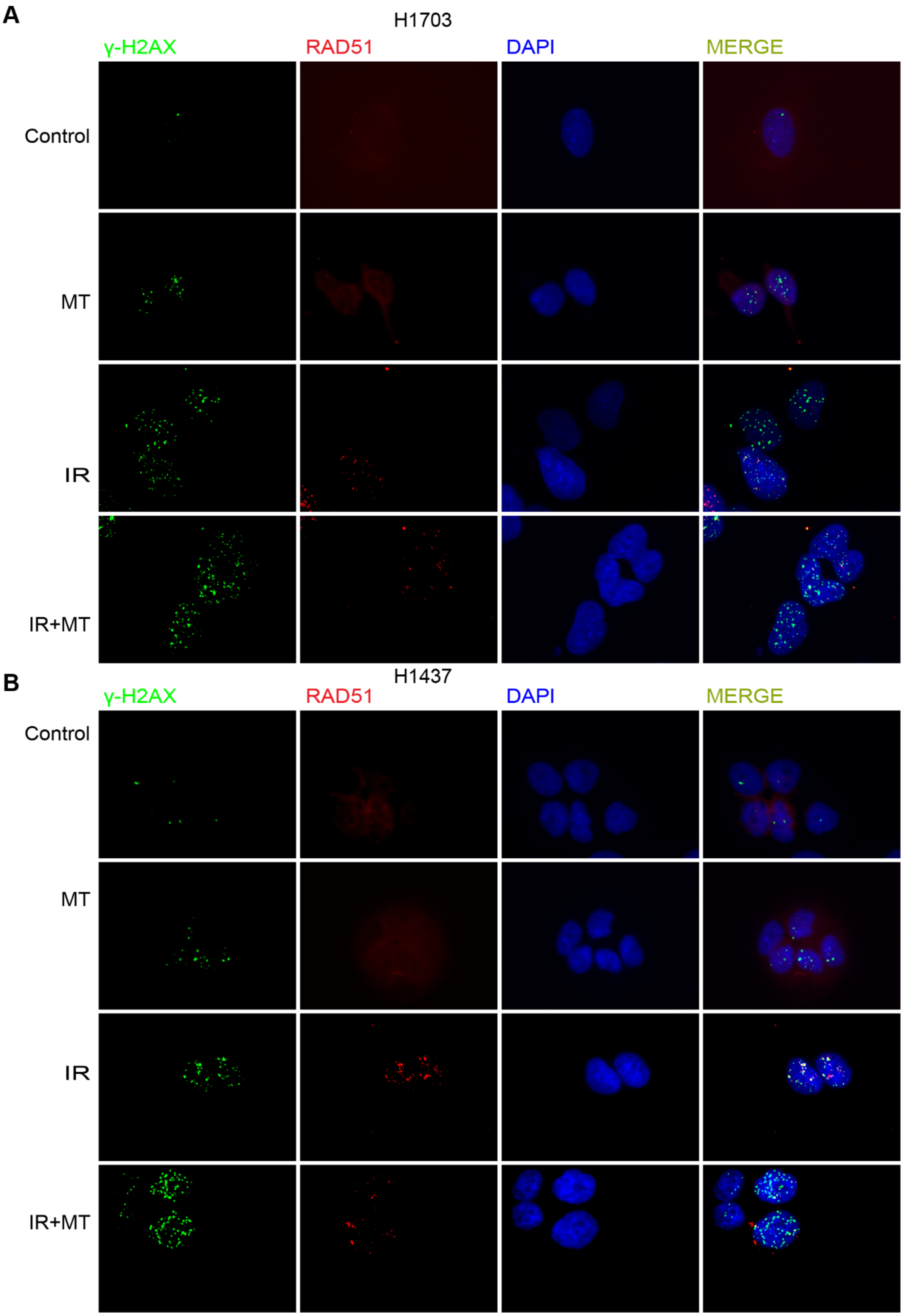
A
H1703
γ-H2AX
RAD51
DAPI
MERGE
Control
MT
IR
IR+MT
B
H1437
γ-H2AX
RAD51
DAPI
MERGE
Control
MT
IR
IR+MT

Figure S5. Melatonin enhances IR sensitivity through suppression of TRIP13.

Representative images of ***(A)*** *H1703 cells and* ***(B)*** *H1437 cells of control, MT, IR and IR with MT treatment stained for γ-H2AX (green) and RAD51(red). Cells were counterstained with DAPI (blue) to visualize nuclei.*

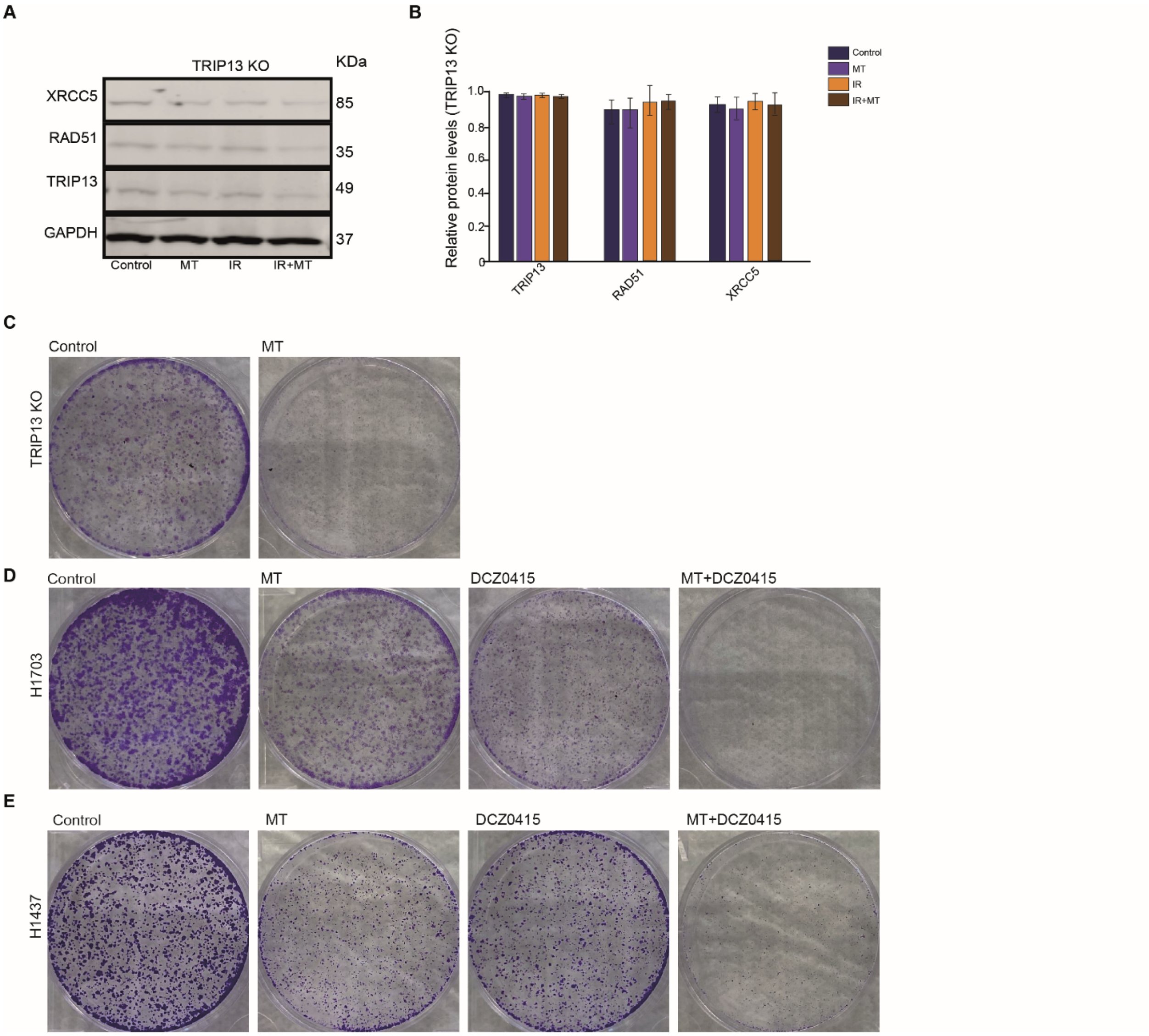

***Figure S6**. Additive Improvement of Anti-Proliferative Effects by the Melatonin Combination DCZ0415*

(A) *Expression of XRCC5, RAD51 and TRIP13 levels of control, MT, IR and IR with MT treated H1703-KO cells.* ***(B)*** *XRCC5, RAD51 and TRIP13 proteins level quantified relative to GAPDH of control, MT, IR and MT+IR treated H1703-KO cell.* ***(C)*** *Representative images of TRIP13-KO cells with and without 1mM MT treatment from clonogenic survival assays. Representative images of* ***(D)*** *H1703 and* ***(E)*** *H1437 cells; control or treated with MT, DCZ0415 or MT+DCZ0415 from clonogenic survival assays.*
